# Draft Genome Sequence of *Acinetobacter* sp. Strain BMW17, a Cellulolytic and Plant Growth-Promoting Bacterium Isolated from the Rhizospheric Region of *Phragmites karka* of Chilika Lake, India

**DOI:** 10.1128/genomeA.00395-16

**Published:** 2016-06-30

**Authors:** Samir R. Mishra, Lopamudra Ray, Ananta Narayan Panda, Neha Sahu, Sonal S. Xess, Sudhir Jadhao, Mrutyunjay Suar, Tapan Kumar Adhya, Gurdeep Rastogi, Ajit Kumar Pattnaik, Vishakha Raina

**Affiliations:** a School of Biotechnology, KIIT University, Bhubaneswar, Odisha, India; b Wetland Research and Training Center, Chilika Development Authority, Department of Forest and Environment, Bhubaneswar, Odisha, India; c Bionivid Technology Private Limited, Kasturi Nagar, Bangalore, India

## Abstract

We report the 3.16 Mb draft genome of *Acinetobacter* sp. strain BMW17, a Gram-negative bacterium in the class of *Gammaproteobacteria*, isolated from the rhizospheric region of *Phragmites karka*, an invasive weed in Chilika Lake, Odisha, India. The strain BMW17^T^ is capable of degrading cellulose and is also an efficient plant growth promoter that can be useful for various phytoremedial and commercial applications.

## GENOME ANNOUNCEMENT

*Acinetobacter* species are Gram-negative, aerobic, nonfermenting, nonfastidious, nonmotile, catalase positive, oxidase negative bacteria that belong to the class of *Gammaproteobacteria*. Several species of *Acinetobacter* have attracted interest due to their ubiquitous nature and ability to metabolize a diverse range of compounds (1–3). Chilika Lake is situated between 19°28′ and 19°54′ N latitude and 85°05′ and 85°38′ E longitude. *Phragmites karka* (a common reed) forms monoculture dense patches in Chilika Lake and is considered to be a highly invasive weed (4, 5).

The microbial communities associated with the submerged parts and rhizospheric region of these macrophytes largely contribute toward overall biogeochemical cycling, and thus play an important role in nutrient cycling in the lagoon ecosystem. The associated bacterial community composition plays a major role in various ecosystem processes and produces many plant growth-promoting compounds, which facilitates the growth of these macrophytes in estuarine environments (6, 7). Moreover, these microbial communities also adapt to a broad range of tolerance against different environmental factors such as pH, temperature, and salinity in brackish water ecosystems. In this study, we report the draft genome sequence of the cellulolytic and plant growth-promoting *Acinetobacter* sp. strain BMW17, isolated from the rhizospheric region of *Phragmites karka*, dense patches of which are located at the Bhaseramundia sampling station of Chilika Lake (19°78224′ N and 85°30041′ E) using the dilution plating technique at 30°C in Zobell’s Marine Broth (HiMedia, India). The strain BMW17^T^ is a Gram negative, cellulase-producing bacterium with optimal growth at temperature 30°C and pH 7.2.

Genomic DNA was extracted using the GNOME kit (MP Biomedicals, Santa Ana, CA, USA). The genome sequence of strain BMW17^T^ was sequenced using the Illumina MiSeq sequencing platform. The data generated was assembled using Velvet assembler (v1.2.10.) (8) and resulted in 113 contigs, a total of 3,560,547 bp, and a *N*_50_ contig size of 98,448 bp. The estimated complete genome size was 3.19 Mb, with a G+C content of 41.40%. Genome annotation was performed using the Rapid Annotation using Subsystems Technology (RAST) server (9, 10), which predicted a total of 3,019 protein coding sequences, 79 pseudogenes, 76 tRNAs, and 3 rRNA clusters. The taxonomy identification was performed using EzTaxon and MEGA6, which identified *Acinetobacter* sp. strain BMW17 as the putative species per 16S rRNA sequence homology. PHAST analysis (11) revealed a putative intact phage integrated in the genome with a length of 54.4 Kb, 44 protein coding sequences, and a G+C content of 41.67%.

Genes for plant growth-promoting characteristics and cellulose degradation are present, corroborating results from RAST annotations which identified various gene clusters for 1-aminocyclopropane1-carboxylate deaminase activity, auxin biosynthesis, nitrogen, metabolism, quorum sensing and biofilm synthesis, siderophore production, phosphorous solubilization, various antibiotic resistance gene clusters and resistance to heavy metals (arsenic, cobalt, zinc, cadmium, chromium), protein degradation, carbohydrate degradation, degradation of aromatic compounds, stress regulation genes, PHB metabolism, lipid metabolism, and sulfur metabolism. In conclusion, *Acinetobacter* sp. strain BMW17 is a promising candidate as an inoculant to stimulate phytoremediation and also for degradation of cellulosic wastes.

### Nucleotide sequence accession numbers.

This whole-genome shotgun project has been deposited in DDBJ/EMBL/GenBank under the accession no. LSOD00000000. The version described in this paper is the first version, LSOD01000000.
